# Temporal relations in hormone-withdrawal migraines and impact on prevention- a diary-based pilot study in combined hormonal contraceptive users

**DOI:** 10.1186/s10194-017-0801-7

**Published:** 2017-08-25

**Authors:** Gabriele S. Merki-Feld, Gina Epple, Nina Caveng, Bruno Imthurn, Burkhardt Seifert, Peter Sandor, Andreas R. Gantenbein

**Affiliations:** 1 Department of Reproductive Endocrinology, University Hospital Zürich, Frauenklinikstrasse 10, CH - 8091, Zürich, Switzerland; 2 Epidemiology, Biostatistics and Prevention Institute, Department of Biostatistics, University of Zürich, Zürich, Switzerland; 3 Neurorehabilitation Rehaclinic Bad Zurzach, Zürich, Switzerland

**Keywords:** Menstrually related migraine, Contraception, Estrogen-withdrawal headache, Migraine without aura, Combined pill, Migraine prevention

## Abstract

**Background:**

Menstrually related migraine (MRM) in the hormone-free interval (HFI) of combined hormonal contraceptives (CHC) are according to the ICHD definition also estrogen withdrawal migraines (EWH). MRMs are less responsive to acute medication. Therefore short-term prevention, initiated 1–2 days before onset of the anticipated bleeding and continued for 6 days, is recommended. Such a long prophylactic triptan use might increase the risk for medication overuse headache in women suffering in addition from non-menstrual migraines. In CHC users onset of hormone decline is predictable. It is however unknown, whether the EWHs are rather associated with onset of hormone withdrawal or onset of bleeding. Improved understanding of this relation might contribute to better define and shorten the time interval for prevention.

**Methods:**

For this observational diary-based pilot study we collected data from daily conducted headache diaries of CHC users with MRM in at least two of three cycles, visiting our clinic from 2009 to 2015. We analyzed frequency of migraines for each hormone free day, onset of migraine, onset of bleeding and the relation of migraine to onset of bleeding in the 7-day period following estrogen withdrawal. We identified in addition the onset of migraine attacks lasting more than 1 day (episodes).

**Results:**

Forty patient charts met the inclusion criteria, what allowed us to analyze 103 cycles. The mean number of migraine days in the HFI was 2.2 ± 1.6. Migraine started typically on days 1–5 and bleeding on days 3–5. In relation to first day of bleeding, migraines started on days −1 to 4. Almost half of the migraine attacks lasted longer than 24 h, despite the use of rescue medication.

**Conclusion:**

MRM in CHC users starts on bleeding days −1 to 4, what differs from findings in the natural cycle. Referring to the HFI interval migraine started mostly on days 1–5. According to these data, it seems to be reasonable to initiate short-term prevention at the last day of pill use or the first day of the HFI and continue for 5 days.

## Background

In women migraine prevalence peaks during reproductive years [1]. Menstruation is a significant risk factor for migraine with attacks most likely to occur between 2 days before the onset of menstruation and the first three days of bleeding. The pathophysiology of menstrual attacks involves estrogen withdrawal and potentially abnormal release of prostaglandins triggered by the end-cycle drop in estrogen levels [2, 3]. In addition to menstrually related migraine (MRM) the ICHD-classification defines estrogen withdrawal headaches (EWH) as migraines arising in women using exogenous estrogen daily for three or more weeks followed by an interruption in which migraine develops within five days after the last estrogen intake [4]. In general MRMs last longer, are more severe, more disabling and less responsive to acute treatment [5–9]. Short-term prevention therefore is an important approach for treatment. Most authors recommend to start preventive therapy two days before the onset of bleeding [10]. However, as in the natural cycle onset of bleeding is highly variable the starting day for prophylactic medications is difficult to predict. Too early start results in unplanned and unforeseeable long use of these pain medications. The latter can result in medication overuse headaches, in those suffering in addition from non-menstrually related migraines. In combined hormonal contraceptive (CHC) users however, the day of estrogen withdrawal is clear, what might facilitate the identification of an optimal interval for short-term prevention. It has however never been investigated, if onset of EWH in CHC users is more related to the onset of bleeding or the onset of the hormone-free time. While in the natural cycle the association of bleeding and migraine has been defined as MRM (days −2 to day 3), the time interval with higher probability for EWH in relation to bleeding in CHC users has never been studied. In contrast to the smooth decline of estrogen levels, the hormone decline in CHC users processes more abrupt and from higher estrogen levels. This might affect onset and course of the following headache. Most studies related to MRM characteristics and treatment do not differentiate women with migraine in the natural cycle and from CHC users experiencing EWH [6, 7, 11–14]. The mixture of different headache types in studies potentially impacts precision of conclusions. With the present study, we aimed to identify the optimal interval for use of preventive migraine agents in CHC users with EWH. For this purpose we identified in addition to migraine days in the pill-free interval, the relationship between migraine onset and day of last pill intake on one hand and the first day of bleeding on the other hand.

## Methods

Patient charts of all female migraineurs were searched to identify premenopausal CHC users, who experienced any migraine in the hormone-free interval (HFI) and visited our clinic for hormonal migraines (University Hospital Zürich) from 2009 to 2015. All charts include headache diaries, which are conducted prospectively over a period of three cycles, before patients come in for their first visit and prior to any intervention. This procedure allows us to identify the type of migraine, especially MRM and EWH according to ICHD criteria. The in the retrospect analysed diaries include daily information on migraine/headache occurrence, pain intensity, use of acute medication, use of hormones and vaginal bleeding. For ethical reasons diaries were anonymized before evaluation and only the main investigator had access to the key and all data. To comply with the ICHD criteria we included only CHC users with EWH in at least two of three cycles. A further criterion of inclusion was use of the CHC in the typical regimen of 21/7 days. Charts were excluded if women used other types of hormonal therapy including progestin-only contraceptives, CHC regimen different from 21/7 and reported headaches in only one of three withdrawal periods. Furthermore charts were excluded if women had not yet used their CHC for at least 6 months.

According to the ICHD definition migraine lasts from 4 to 72 h. As MRM migraines tend to last longer, we evaluated both, migraine days and migraine episodes (lasting >24 h). To identify onset of bleeding, migraine days, the first day of migraine and the start of migraine episodes we collected data during days 1–7 of the HFI. To explore the vulnerable time frame for migraine onset in relation to the withdrawal bleeding we choose the interval of days −3 to day 5, which allowed us to exclude that we might miss an association by just including two days before bleeding and the first three bleeding days. A similar procedure has been used earlier by MacGregor [15].

### Statistical analyses

The programs IBM SPSS Statistics 22 and Excel 2013 were used for the statistical evaluation of this project. Continuous demographic and clinical characteristics are presented as means and standard deviations (SD). Frequencies and percentages were used to describe migraine days, migraine episodes and bleeding days over the described time intervals. For these comparisons chi-square test was used.

## Results

**Fig. 1:**
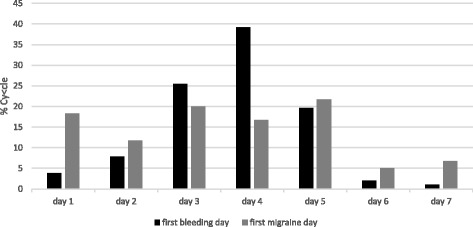
First day of bleeding and first migraine day in the seven days of the hormone-free interval of combined hormonal contraceptive users

**Fig. 2:**
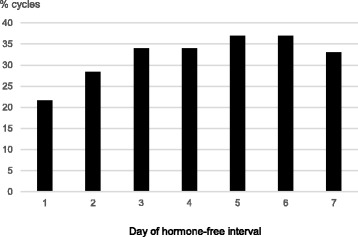
Frequency of migraine days in the seven days of the hormone-free interval of combined hormonal contraceptive users

**Fig. 3:**
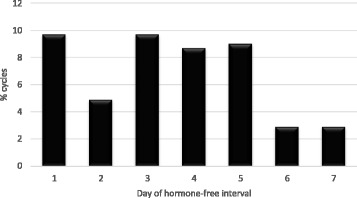
First migraine episode at each day of the hormone-free interval of combined hormonal contraceptive users

**Fig. 4:**
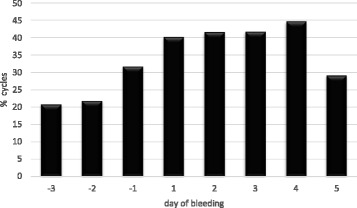
Migraine attacks in combined hormonal contraceptive users in relation to start of bleeding during days −3 to day 5

We identified 92 CHC users out of 177 patient charts from all premenopausal female migraineurs visiting our clinic between 2009 and 2015. Forty met all inclusion criteria and were eligible for evaluation. From the excluded patients 27 had not conducted complete diaries over 3 cycles or had used CHC continuously (long-cycle), 11 patients used CHC regimen different from 21/7 and 14 patients had suffered from migraine only in one out of three cycles. As at present there is no evidence that CHC-related estrogen-withdrawal headache is influenced by the progestin-type, CHC with all types of progestins were included. In the present study these were levonorgestrel, desogestrel, drospirenone, gestoden and cyproterone-acetate. The daily ethinylestradiol dosage ranged from 20 to 30 μg. In total data of 103 cycles were eligible for analyses. Twenty-three patients experienced migraine in all three cycles, while 17 patients suffered from migraine in only two out of three cycles. All patients had started their CHC more than 6 months before inclusion. Mean age of the forty included patients was 29.1 (18–40) years. The mean number of migraine days during the HFI was 2.2 ± 1.6 (mean ± SD). Migraine without aura was more common than migraine with aura in the history of the patients with a total of 33 patients (82.5%) versus 7 patients (17.5%). Migraine episodes were observed in 49% of all cycles. Most withdrawal bleedings started at day 4 of the HFI (39.2% of cycles) (Fig. 1). In 84.3% of the cycles the first day of withdrawal bleeding was reported on pill-free days 3 to 5. The first bleeding day in the HFI varied one day in 75% of the migraineurs and two days or less from cycle to cycle in more than 90% of the women and insofar was much better predictable in comparison with the natural cycle. The frequency of all migraines at each day of the HFI is demonstrated in Fig. 2. It increased from day two onward and was above 32% on days 3–7. The days with highest percentages of migraine attacks were days 5 and 6 with 36.9% of cycles each closely followed by the days 3 and 4 (34%) and day 7 (33%). Migraine onset in the HFI was mostly on days 1–5 (Fig. 1). Alike the start of migraine episodes was typically at days 1–5 and rarely during days six and seven (Fig. 3). In relation to the start of withdrawal bleeding migraine frequency started to rise at day one before bleeding (31.7% of the cycles) (Fig. 4). In more than 40% of the analyzed cycles migraine attacks occurred on bleeding days 1, 2, 3 and 4.

## Discussion

In the present study we analyzed the onset and course of single migraine days and migraine episodes during the HFI in CHC users. In the latter migraine could be associated with either, the sharp hormone decline immediately after stopping the pill or with the onset of withdrawal bleeding some days later. To optimize the duration of short-term prevention and avoid medication overuse headaches in women suffering in addition from non-menstrual related attacks this issue is of relevance. Analyses of headaches and bleeding over the seven hormone-free days revealed that bleeding mostly starts during days 3–5 and onset is in contrast to the natural cycle highly predictable. The first migraine attack occured on days 1–5 (each 12–20% of cycles), indicating that prevention has to start earlier than the expected withdawal bleeding (Fig. 1). Migraine start at days 1–5 is in accordance with the 5-day period defined for estrogen-withdrawal headaches by the ICHD. Most migraine days were observed on days 3–7 (each in more than 33% of cycles, Fig. 2). In relation to withdrawal bleeding most migraines occurred between day −1 and day 4. There was a twofold frequency of migraine attacks on bleeding days 1–4 in comparison to day −2 in our study. This differs from previous studies exploring MRM in the natural cycle, which identified peaks of attacks from bleeding days −2 to day 3 and is exactly one day later, in comparison to the ICHD definition [6, 16]. The abrupt estrogen decline in women stopping use of exogenous applied estrogens together with its effects on bleeding could explain this difference. Also preliminary this finding suggests that using the same time frame for both, MRM in the natural cycle and after use of artificial estrogens in CHC users might be too imprecise.

MRMs have been found to be longer lasting, more severe and refractory to abortive medications [6, 17]. As the majority of our study participants suffered from both MRM and non-menstrually related migraine attacks we cannot exclude that some of the migraine attacks during the HFI are only randomly associated with hormone withdrawal. Separate analyses of longer lasting attacks might provide a better approach to identify truly hormone- withdrawal associated migraines. Episodes (attacks lasting >1 day) in our study were observed in 49% of 103 cycles with a mean duration of more than 3 days, in spite of use of rescue medication. The high rate of long lasting episodes of EWH in CHC users indicates that these headaches similar as the MRM in the natural cycle, are also long lasting and less responsiveness to rescue medication [6, 7]. Most of these episodes started on days 1–5 of the HFI, as well.

To our knowledge this is the first study analyzing the course of migraine during the hormone-free interval in CHC users, which includes in accordance with the ICHD criteria only women with EWM in at least two of three cycles. One study, including women with MRM in the HFI reported highest pain scores at HFI day 4 in eleven CHC users, but was limited with regard to the low number of CHC users and the observation period of only one cycle [18]. Lieba-Samal et al. investigated prevalence of headache and migraine in CHC users in association with their withdrawal bleeding [19]. They found that CHC users had a twofold hazard ratio for migraines at bleeding days 1–3, but not at days −1/−2. Inclusion criteria comprised at least one day of migraine per month, without consideration of the HFI. We in addition to bleeding days 1–3 observed more migraine attacks at days -1and day 4. Our daily analyses of headache days over days −3 to day 5 is not comparable with the mentioned interval analyses and much more exact for the objective. Numerous studies demonstrated that MRM attacks last longer in comparison to non-menstrual migraine attacks [7, 20]. Characteristics of migraine attacks are highly variable not only among patients but also within the same patient [21]. For preventive therapy of EWH the majority of medications have been tested to be started two days before the expected onset of menstrual flow and to be continued for 6 days [10, 22–24]. The low predictability of bleeding and hormone withdrawal in the natural cycle makes it difficult to apply to such a regimen and frequently results in treatment periods of 8 and more days [15]. In women with additional non-menstrual attacks this may cause an unacceptable high use of rescue medications. Our data demonstrate that first day of migraine and migraine episodes rarely occur later than day 6 of the HFI. Referring to our results it appears to be reasonable to initiate short-term prevention in CHC users at the last day of pill intake or the first day of the HFI and continue for 5 days. Even if withdrawal bleeding in CHC users is highly predictable, our data indicate that use of the HFI as reference point is the better choice.Starting prevention at the last day of pill intake would also be optimal to prevent most of the episodes, which are very disabling, long-lasting and difficult to treat.

Strengths of the current study include the exact diagnosis of menstrually related migraine according to the ICHD-criteria, the accurate prospectively conducted daily diaries, the inclusion of only women with the typical 21/7 CHC regimen and the headache documentation over three cycles. Analyzing first days of migraine episodes increases the probability to identify the onset of true menstrually related attacks with low responsiveness to abortive medications. Analyses of the reproducibility of the first bleeding day over several cycles might contribute to optimize treatment in some women. A potential limitation is the evaluation of the diaries in the retrospect. We cann’t exclude that inclusion of CHC containing different ethinylestradiol dosages (20 μg and 30 μg) might exert an impact on our results. Ethinylestradiol is much more potent than the naturally released estradiol. With both CHC dosages, estrogen withdrawal occurs from higher estrogen levels and is much more abrupt in comparison to the natural cycle. Therefore we do not think that the small differences in ethinylestradiol dosage are relevant in the context of this study. The number of more than 100 evaluated cycles is sufficient for a pilot study, but the results have to be confirmed in prospectively conducted studies including more cycles. Data from women attending a specialist migraine office cannot always be extrapolated to the general population.Even taking into account these limitations we consider the present data as useful to clinicians who treat CHC users with EWH and have to define the time frame for short-term prevention. In this context it has to be mentioned that treatment of migraine in CHC users should not only imply rescue medications or prophylactic agents, but also the consideration to change to a progestin- only method. These are not only safer but may reduce migraine frequency and intensity as well [25–32]. Use of CHC in extended cycles regimen is another approach to improve withdrawal migraine but is not recommended, as risk of breast cancer with those pill regimen is unknown and risk for ischemic stroke could further increase [16, 22, 30]. For women with MRM a multidisciplinary approach combining neurological and gynaecological consultations might be of advantage [33].

## Conclusions

In CHC users migraine mostly starts on bleeding days −1 to day 4, what differs from findings in the natural cycle. Referring to the HFI interval most migraines and migraine episodes start at days 1–5. Around 50% of the migraine attacks last more than 24 h. Short-term prevention could contribute to better management of these episodes and should be initiated at the last day of pill use or the first day of the HFI and be continued for 5 days.

## Abbreviations

CHC: Combined hormonal contraceptives
EWH: Estrogen withdrawal headache
HFI: Hormone-free interval
MRM: Menstrually related migraine
SD: Standard Deviation.
